# Trends in HIV incidence following scale-up of harm reduction interventions among people who inject drugs in Kachin, Myanmar, 2008–2020: analysis of a retrospective cohort dataset

**DOI:** 10.1016/j.lanwpc.2023.100718

**Published:** 2023-02-27

**Authors:** Anna L. McNaughton, Jack Stone, Khine Thet Oo, Zaw Zen Let, Mar Taw, Minn Thit Aung, Aung Myo Min, Aaron G. Lim, Ernst Wisse, Peter Vickerman

**Affiliations:** a Population Health Science, Bristol Medical School, University of Bristol, Bristol, UK; b Community Support Worker, Kachin, Myanmar; c Médecins du Monde, Kachin, Myanmar; d Médecins du Monde, Paris, France

**Keywords:** HIV, People who inject drugs, Opioid agonist therapy, Myanmar, Burma, Needle and syringe provision

## Abstract

**Background:**

People who inject drugs (PWID) in Kachin, Myanmar, have a high HIV prevalence (>40%), but there is no data on incidence. We used HIV testing data from three harm reduction drop-in centres (DIC) in Kachin (2008–2020) to determine HIV incidence trends among PWID and associations with intervention uptake.

**Methods:**

Individuals were HIV-tested at first DIC visit and periodically thereafter, during which demographic and risk behaviour data were collected. Two DIC provided opioid agonist therapy (OAT) from 2008. Monthly DIC-level needle/syringe provision (NSP) data was available from 2012. Site-level 6-monthly NSP coverage was denoted low, high, or medium if it was below the lower quartile, above upper quartile, between these quartiles of provision levels over 2012–2020, respectively. HIV incidence was estimated by linking subsequent test records for those initially testing HIV-negative. Associations with HIV incidence were examined using Cox regression.

**Findings:**

Follow-up HIV testing data was available for 31.4% (2227) of PWID initially testing HIV-negative, with 444 incident HIV infections during 6266.5 person years (py) of follow-up. Overall HIV incidence was 7.1 per 100 py (95% confidence interval 6.5–7.8), which decreased from 19.3 (13.3–28.2) in 2008–11 to 5.2 per 100 py (4.6–5.9) in 2017–20. In the full PWID incidence dataset after adjustment for various factors, recent (≤6 weeks) injecting (aHR 1.74, 1.35–2.25) and needle sharing (aHR 2.00, 1.48–2.70) were associated with higher incidence, while longer injection careers were associated with reduced incidence (aHR 0.54, 0.34–0.86, for 2–5 yrs vs <2 yrs). In a reduced dataset including data on OAT access and NSP coverage (2012–2020 for two DIC providing OAT), being on OAT during follow-up was associated with reduced HIV incidence (aHR 0.36, 0.27–0.48, compared to never being on OAT) as was high NSP coverage (aHR 0.64, 0.48–0.84, compared to medium syringe coverage).

**Interpretation:**

Although HIV incidence is high among PWID in Kachin, data suggests it has decreased since the scale-up in harm reduction interventions.

**Funding:**

US NIH, Médecins du Monde.

**Research in context**

**Evidence before this study**

Accurate estimations of HIV incidence among people who inject drugs (PWID) are important for developing intervention strategies for achieving HIV elimination. We reviewed studies on HIV incidence among PWID published since 2000. Whilst a good proportion of the studies (11 out of 46 studies) were from low- or middle-income countries (LMICs), these came from only seven LMIC and were mostly from low-incidence settings, with just four studies from Ukraine, India and Pakistan having a HIV incidence greater than 5.0 per 100 person years (py), and only four studies with more than 50 documented seroconversions. Other studies from the region provide HIV incidence estimates among PWID for Thailand, Vietnam, China, India and Bangladesh. Reductions in HIV acquisition risk from being in contact with harm reduction interventions, including opioid agonist therapy (OAT) and clean needle and syringe provision (NSP), are well evidenced in high-income settings but evidence of their impact in LMICs is lacking. The literature reviewed identified two studies in LMICs that assessed the impact of OAT and NSP, both in India. In these studies, OAT had limited impact and NSP was found to be associated with a higher risk of HIV infection, although this may be due to those attending the service having high injection frequencies. Previous systematic reviews of the effectiveness of OAT and NSP included no studies from LMICs.

**Added value of this study**

Kachin is a largely rural conflict-affected area in North Myanmar, especially affected by injecting drug use and HIV. This is the first study to estimate HIV incidence among PWID in Myanmar. Using routine data on HIV testing and other factors collected from harm reduction services run by Médecins du Monde (MdM) in Kachin, we generated a large longitudinal cohort of PWID that were initially HIV-negative (n = 2227), capturing 6266.5 person years (py) of follow-up and 444 incident HIV infections. Findings from the study indicate that HIV incidence in the region is high but has declined in recent years, from 19.3 per 100 person years (py) (13.3–28.2 py) in 2008–11 to 5.2 py (4.6–5.9 py) in 2017–20. Harm reduction interventions in the region scaled up considerably over the 12-year study period, with the numbers of needles distributed per month tripling and 67.4% of eligible PWID attending the MdM harm reduction services being on OAT by the end of the study. Despite the socio-political challenges in the region, our multi-variable regression analysis suggested that being on OAT was associated with a two-thirds reduced risk of HIV acquisition (aHR 0.36, CI 0.27–0.48, compared to never being on OAT) while high NSP coverage was associated with a one-third reduced risk (aHR 0.64, CI 0.48–0.84, compared to medium NSP coverage).

**Implications of all the available evidence**

Our study highlights the importance and utility of using routinely-collected service use and HIV testing data from targeted HIV prevention interventions for monitoring incidence trends among PWID. Whilst this study focused on HIV among PWID in Kachin, consideration should be given to using this approach in other settings, both for HIV and other infections affecting PWID populations and other key populations. Findings from this study provide evidence of the substantial impact that OAT and NSP can have in LMICs when high levels of provision are achieved. This underscores the importance of offering widespread OAT and NSP among PWID populations, regardless of context, and of using similar routine collected data to evaluate the impact of these interventions. This highlights the utility of ensuring high quality routine data collection by HIV prevention services, and the importance of including other variables in such datasets to enable more detailed analyses of how risk factors influence incidence.

## Introduction

An estimated 1.7 million persons are living with HIV (PLHIV) in the WHO South East Asia region (SEARO),^1^ with 2015 estimates suggesting 225,000 PLHIV in Myanmar.^2^ HIV infections are disproportionately concentrated among key populations in SEARO, with UNAIDS estimating that people who inject drugs (PWID), men who sex with men and sex workers contribute 94% of new HIV infections in the region.^2^^,^^3^ Globally, 2.8 million PWID are thought to be living with HIV, with 605,000 in SEARO.^4^

Myanmar is the second-largest opium producing region globally, with most cultivation occurring in the Shan and Kachin States.^5^ HIV prevalence among PWID is high in Myanmar (35% in 2017), with Kachin reporting a particularly high HIV prevalence (47.2% in 2017).^6^^,^^7^ Kachin State reports the highest prevalence of HIV in the country (2.8%; national prevalence is 0.6%^6^), and is estimated to contain 23% of all PWID in Myanmar.^6^ Jade mining, historical opium use, illicit drug cultivation and trafficking may contribute to the heightened drug use in Kachin^8^^,^^9^.

Interventions for reducing HIV transmission among PWID include needle and syringe provision (NSP), opioid agonist therapy (OAT) and antiretroviral therapy (ART). NSP in Myanmar started in 2003, while OAT was initiated in 2006.^10^ Since 2014, the number of needles/syringes distributed in Myanmar has increased 2.5-fold to 33 million needles per year by 2018 and the number of people on OAT has increased similarly, with approximately 16,000 PWID on OAT in 2018.^11^ ART provision has also scaled up over recent years, with Myanmar adopting the WHO recommendation to treat all PLHIV in 2017.^2^

Médecins du Monde (MdM) has provided harm reduction (HR) services in Myanmar since 1994.^12^ Their services in Kachin initiated in 1996,^12^ including three HR intervention-sites in Myitkyina, Mogaung and Hopin. These interventions provide outreach NSP and three drop-in centres (DIC), which provide voluntary HIV counselling and testing (VCT), OAT and ART. MdM have achieved considerable scale-up of services (Supplementary Fig. S1). Other HR providers are also active in Kachin, with MdM distributing 17.7% of all syringes in 2019.^13^

Ongoing improvements in HR services in Kachin have been achieved despite difficulties resulting from the ongoing conflict between the Kachin Independence Army (KIA) and Burmese Military since mid-2012, the effects of which are uncertain. Actions of an anti-drug vigilante group, the ‘Pat Jasan’,^12^^,^^14^ also led to forced detoxification camps and disruptions in HR services, including a forced closure of the Mogaung DIC for 6 months over 2016–2017. Lockdowns resulting from the SARS-CoV-2 pandemic (2020) and the 2021 Government coup also caused reductions in HR services (Supplementary Figs. S1, S3 and S4).

Although evidence suggests that OAT and NSP can reduce the risk of HIV acquisition, most data is from high-income countries.^15^, ^16^, ^17^ Little empirical data also exists on the incidence of HIV among PWID from lower or middle-income countries (LMIC). In this study, we utilised routine collected individual-level data on HIV testing and intervention access from three MdM DIC in Kachin, over 2008–2020 to determine trends in HIV incidence among the PWID population, and to evaluate whether uptake of OAT and/or scale-up in NSP were associated with reduced HIV incidence.

## Materials and methods

### Data

MdM routinely collect data from DIC clients when presenting for HIV testing. As well as PWID, the DIC provides services for people who use drugs and their sexual partners. Clients are given a unique anonymous code at their first visit which is used thereafter. A standardised form is used to collect data from clients, including: date of program registration, date of visit, date of birth, sex, population risk group, drug use (substance and method of use), duration of drug use, syringe sharing in previous 6 weeks, and HIV test result. HIV testing is performed using the SD-bioline rapid test. Client-level data was available for 2008–2020 in Myitkyina and Mogaung and 2011–2020 for Hopin. Information on OAT enrolment was only available in Mogaung and Hopin. In Myitkyina, OAT provision was through a different provider. Data on the number of syringes exchanged was only available at the DIC level for each month over 2012–2021. People were included in this analysis if they had ever been tested for HIV.

Other DIC service provision data was available over 2012–2021, including the monthly number of visits to the services, number of people tested for HIV, and number of individuals newly initiated on ART and OAT. ART was provided at all DIC.

### Variables and definitions

The prevalence of HIV among clients was assessed using an individual's first test result, which generally occurred at enrolment. The primary study outcome were HIV seroconversions, which were identified among the sub-group of individuals that initially tested HIV-negative and had subsequent HIV test results, denoted as the ‘***incidence dataset***’. As there were frequently long intervals between re-testing (often >1 yr), a positive HIV test result indicates that an individual was infected sometime between their last negative result and first positive result. The estimated time for each seroconversion was assumed to be half-way between these test results. The length of follow-up for each HIV seroconversion was the time from the initial HIV-negative result to the estimated time of seroconversion, while for an individual without seroconversion it was the time from the initial HIV-negative test result to their last recorded HIV-negative result.

This analysis considered the following factors that may affect HIV incidence: time period (2008–2011, 2012–2016, 2017–2020); DIC site; age of individual (<25 years, ≥25 years); sex (male/female); length of follow-up in DIC (<3 years, ≥3 years); and population risk group. Population risk groups were defined based on their drug use at enrolment, with PWID defined as people having injected drugs in the past year, people who use drugs (PWUD) defined as people having used drugs but not by injection in the past year, and non-drug users (non-PWUD). Non-PWUD attending the DIC were generally sexual partners of PWID or PWUD, although a small proportion were general population or children. PWUD and non-PWUD who reported injecting drug use at a specific follow-up were reclassified as PWID thereafter, while PWID remained as such even if they reported periods of non-injecting.

Among PWID, we considered additional individual-level time varying characteristics including duration of injecting drug use (<2 years, 2–5 years, 5–10 years, ≥10 years), whether injected drugs in last 6 weeks (yes/no), polydrug use in last year (use of >1 type of drug, yes/no), syringe sharing in last 6 weeks (yes/no), and whether they were currently on OAT. Injecting risk factor questions were only asked of PWID thought to be higher risk. Data was available on the date individuals initiated OAT, with DIC clients assumed to be taking OAT until recorded otherwise. OAT status was defined by four categories: on OAT during follow-up but not yet initiated, currently on OAT, on OAT during follow-up but not anymore, and never on OAT during follow-up. We included stratification of individuals into those that have not yet started OAT or have stopped OAT to assess whether these individuals have reduced risk unrelated to taking OAT. We also considered the number of syringes distributed by each DIC over 6-monthly periods as a site-specific exposure variable defined as low, medium, or high coverage, based on whether the number of syringes distributed in that period was below the lower quartile, between the quartiles or above the upper quartile of what was distributed in each period, respectively (Supplementary Table S1).

### Analysis

Descriptive analyses were undertaken to characterise the incidence dataset, and how that changed over time. Characteristics of HIV-negative individuals with and without follow-up were compared using Fisher's exact and Kruskal–Wallis tests. Response rates for behaviour-related variables varied; missing data was recorded as ‘no response’, which could denote that the individual was thought to not have that risk factor.

For the incidence dataset, HIV incidence was calculated by dividing the overall number of seroconversions by the total follow-up time. Cox regression analyses were firstly undertaken to determine whether population risk group and sex were associated with HIV incidence in the overall incidence cohort, and then to ascertain whether demographic, risk and intervention factors were associated with HIV incidence among PWID. This second analysis allowed for relevant variables being time varying. Four separate regression models were constructed for PWID. Two considered the full time-period (2008–2020) without including NSP exposure, with model 1 including PWID from all DIC, but not including OAT status because this data was not available for Myitkyina, and model 2 including PWID from just Mogaung and Hopin with OAT status included. The other models were over 2012–2020 so that NSP coverage could be included as a DIC-level exposure variable, with model 3 including all three DIC but not OAT status and model 4 only including Mogaung and Hopin with OAT status included. Recent injection drug use was excluded from models 2 and 4 that included OAT status, while recent needle sharing was excluded from models 3 and 4 that included NSP exposure, as those behaviours are considered mediators for the effect of these interventions.^18^ Unsafe sex in the last 6 weeks was only included in a restricted model (covering 2017–2020) as data was unavailable for other years. A sensitivity analysis was undertaken excluding data from 2020 in model 4 to determine whether closures associated with the SARS-CoV-2 pandemic may affect our results.

Unadjusted hazard ratios (HR) from bivariate models and adjusted HRs (aHR) from multivariable models were estimated. All variables from the bivariate model were included in the multivariable model apart from age, which was co-linear with duration of injection drug use. All confidence intervals (CI) are 95% CIs and p-values are two-sided.

All the analyses were performed using STATA (version 16.1).

### Ethics

All analysed data was collected routinely by MdM through their HR programs in Kachin. Approval for the study was granted by the University of Bristol Faculty Research Ethics Committee, project reference 10266.

### Role of the funding source

The primary funder MdM contributed to the analysis plan and write up. University of Bristol had final responsibility for the analysis, write up and decision to publish.

## Results

### Overall sample characteristics

Over the study period 2008–2020, 20,761 individuals had a first HIV-test at the three DIC (Table 1) with 6689 people tested in Hopin (32.2%), 5973 in Mogaung (28.5%), and 8099 in Myitkyina (39.0%). Most individuals tested for HIV were PWID (67.8%), with PWUD and non-PWUD both accounting for 16.1% of attendees. HIV prevalence at first test among all attendees was 37.1% (95% CI 36.4–37.8%) (Table 1), but was higher among PWID (48.4%; 95% CI 47.6–49.2%) than PWUD (12.4%; 11.3–13.6%) and non-PWUD (14.3%; 95% CI 13.2–15.5%). Supplementary Table S2 includes HIV prevalence among different non-PWID groups. Among PWID, HIV prevalence was highest at the Hopin (49.8%; 95% CI 48.5–51.1%) and Myitkyina (50.3%; 95% CI 48.8–51.8%) DICs, and lower in Mogaung (44.2%; 95% CI 42.6–45.8%). HIV prevalence increased among first HIV-tests of PWID over 2008–2020, especially from 2014, peaking at 58.1% (95% CI 54.8–61.3%) in 2016 (Supplementary Fig. S2).

**Table 1:** Demographic characteristics and HIV prevalence of individuals attending the drop-in centres (DIC) in Kachin for their first recorded HIV tests (2008–2020).

Characteristic	Group	Total (n=)	Proportion (%)	HIV positive (n=)	HIV positive (%)	p-value
	All	20,761	100	7704	37.1 (36.4–37.8)	–
DIC client group	PWID	14,069	67.8	6810	48.4 (47.6–49.2)	<0.001
	PWUD	3352	16.1	416	12.4 (11.3–13.6)	
	Non-PWUD	3340	16.1	478	14.3 (13.2–15.5)	
**Among PWID only**						

Gender	Male	13,849	98.4	6694	48.3 (47.5–62.9)	0.275
	Female	220	1.6	116	52.7 (46.1–59.3)	
Centre	Hopin	4343	30.9	2183	49.8 (48.5–51.1)	<0.001
	Mogaung	3871	27.5	1711	44.2 (42.6–45.8)	
	Myitkyina	5855	41.6	2916	50.3 (48.8–51.8)	
Year of HIV test	2008–11	1532	10.9	694	45.3 (42.8–47.8)	0.036
	2012–16	4526	32.2	2202	48.7 (47.2–50.1)	
	2017–20	8011	56.9	3914	48.9 (47.8–50.0)	
Injected drugs within last 6 weeks	No	4205	29.9	2023	48.1 (46.6–49.6)	0.898
	Yes	6177	43.9	2999	48.6 (47.3–49.6)	
	No response	3687	26.2	1788	48.5 (46.9–50.1)	
Shared needles within last 6 weeks	No	9056	64.4	4147	45.8 (44.8–46.8)	<0.001
	Yes	1433	10.2	928	64.8 (62.2–67.2)	
	No response	3580	25.4	1735	48.5 (46.8–50.1)	
Unsafe sex within last 6 weeks^a^	No	4671	33.2	2231	47.8 (46.3–49.2)	0.469
	Yes	558	4.0	265	47.5 (43.4–51.7)	
	No response	8840	62.8	4314	48.8 (47.8–49.8)	
Reports polydrug use^b^	No	11,069	78.7	5353	48.3 (47.4–49.3)	0.915
	Yes	682	4.8	327	47.9 (44.2–51.7)	
	No response	2318	16.5	1130	48.7 (46.7–50.8)	
Age	Mean (yrs)	30.2 yrs (30.1–30.4)				
	<20	633	4.5	230	36.2 (32.7–40.2)	<0.001
	20–24	3160	22.5	1411	44.7 (42.9–46.4)	
	25–29	3683	26.2	1869	50.7 (49.1–52.4)	
	30–34	2831	20.1	1447	51.1 (49.2–53.0)	
	35–40	1816	12.9	931	51.3 (49.0–53.6)	
	40+	1934	13.8	915	47.3 (45.1–49.5)	
Age at first use	Mean (yrs)	24.6 yrs (24.4–24.7)				
	<20	2727	19.4	1317	48.3 (46.4–50.2)	0.012
	20–24	3187	22.7	1560	48.9 (47.2–50.7)	
	25–29	2102	14.9	1056	50.2 (48.1–52.4)	
	30–34	1099	7.8	528	48.0 (45.1–51.0)	
	35–40	632	4.5	301	47.6 (43.8–51.5)	
	40+	423	3.0	168	39.7 (35.1–44.5)	
	No response	3899	27.7	1880	48.2 (46.7–49.8)	
Duration of injection drug use	Mean (yrs)	5.9 yrs (5.8–5.9)				
	<2	1118	7.9	414	37.0 (34.2–39.9)	<0.001
	2–<5	3967	28.2	1828	46.1 (44.5–47.6)	
	5–<10	3260	23.2	1699	52.1 (50.4–53.8)	
	≥10	2085	15.8	427	53.8 (51.7–55.9)	
	No response	3639	25.9	1743	47.9 (46.3–49.5)	

a Unsafe sex in the last 6 weeks was only reported for some of the DIC clients during 2017–2020, meaning this data was unavailable for the majority of clients.

b Polydrug use was recorded as ‘yes’ for PWID taking multiple different classes of drugs. This was typically heroin or other opiates plus either amphetamines, yama (a mixture of methamphetamine and caffeine widely used in South East Asia) or alcohol. The majority of PWID in the study reported taking either heroin and/or opiates, which were considered the same drug class and recorded as ‘no polydrug use’.

### Characteristics of the incidence dataset

In the whole sample, 13,057 individuals were HIV-negative at their first test, with 3685 of these individuals having further HIV testing to make up the incidence dataset (Supplementary Table S3). The majority were PWID (61.8% at their first HIV-test), with 18.8% being PWUD and 19.4% being non-PWUD. The mean age of individuals in the incidence dataset was 30.9 years [yrs] (95% CI 30.5–31.2 yrs) at their first HIV-test and 22.3% were female. PWID were generally younger than PWUD (mean 29.0 yrs vs 38.4 yrs; p < 0.001) and a similar age to non-PWUD (29.6 yrs). Most PWID and PWUD were male (98.4% and 86.4% respectively), while most non-PWUD were female (93.9%), reflecting that sexual partners of people who use drugs also attend the DIC. At their first HIV-test, recent injecting drug use was reported by 41.3% of PWID and 9.3% reported recent syringe sharing, but 22.2% and 20.7% of PWID recorded no response to these questions, respectively (Supplementary Table S3).

In the incidence dataset for Myitkyina and Hopin, 64.0% of PWID recorded being on OAT at some point during follow-up, with this increasing from 3.4% in 2012 to 68.3% in 2020. The mean follow-up for individuals while on OAT was 2.2 yrs (95% CI 2.1–2.3 yrs), with 6.8% (n = 81) ceasing OAT during follow-up. NSP coverage data indicated that provision tripled over 2012–2021 from a mean of 44,583 needles per month in 2012 to 132,644 in 2021 (Supplementary Fig. S1).

Compared to the full sample of HIV-negative individuals, a greater proportion of PWID in the incidence dataset were first tested in 2008–2011 (Supplementary Table S3) or tested at the Mogaung site (42.2% vs 29.8%, p < 0.001). Although Injecting and sexual risk behaviours did differ between the incidence dataset and the full sample of HIV-negative individuals, the differences were generally small except for a greater proportion of the incidence dataset reporting syringe sharing (9.0% vs 7.0% in the full dataset; Supplementary Table S3).

### HIV incidence

Individuals within the incidence dataset had a mean of 2.7 (95% CI 2.6–2.7) HIV tests over a mean follow-up of 2.4 yrs (95% CI 2.3–2.5 yrs). The total follow-up time was 8976.2 yrs, during which 473 incident HIV infections occurred, giving an overall incidence of 5.3 (95% CI 4.8–5.8) infections per 100 person years (py) (Table 2). Most new HIV infections (93.9%, n = 444) occurred among PWID, giving an incidence of 7.1 (95% CI 6.5–7.8) infections per 100 py among PWID (Table 2), which is higher than among PWUD (1.0; 95% CI 0.6–1.7) and non-PWUD (1.1; 95% CI 0.7–1.8). HIV incidence is also higher in males than females across the entire incidence dataset, although this is primarily due to most PWID being male.

**Table 2:** HIV incidence estimates (including unadjusted hazard ratios [HR]) over the 2008–2020 period among different population groups, and by different stratifications among PWID attending the drop-in centres in Kachin.

Group	Baseline population size (n=)	Total person years of follow-up (yr)	Number of HIV infections (n=)	Rate per 100 py	95% CI	HR (unadjusted)	95% CI	p-value
Total	3685	8976.2	473	5.3	4.8–5.8	n/a		
PWUD	692	1295.2	13	1.0	0.6–1.7	1		
non-PWUD	716	1414.4	16	1.1	0.7–1.8	1.24	0.62–2.48	0.548
PWID	2277	6266.5	444	7.1	6.5–7.8	7.68	4.59–12.85	<0.001
Male	2896	7288.42	447	6.1	5.6–6.7	1		
Female	789	1687.7	26	1.5	1.0–2.3	0.25	0.17–0.38	<0.001
Among PWID only								

Male PWID	2246	6173.4	436	7.1	6.4–7.8	1		
Female PWID	31	93.1	8	8.6	4.3–17.2	1.21	0.60–2.43	0.601
Age <25 yrs	777	1587.1	152	9.6	8.2–11.2	1		
Age ≥25 yrs	1500	4679.5	292	6.2	5.6–7.0	0.69	0.60–0.83	<0.001
Hopin	773	2144.8	139	6.5	5.5–7.7	1		
Mogaung	962	3076.8	186	6.0	5.2–7.0	0.95	0.76–1.18	0.646
Myitkyina	542	1044.9	119	11.4	9.5–13.6	1.73	1.35–2.21	<0.001
2008–2011	365	139.6	27	19.3	13.3–28.2	1		
2012–2016	975	1253.7	165	13.2	11.3–15.3	0.66	0.44–1.00	0.053
2017–2020	937	4873.3	252	5.2	4.6–5.9	0.28	0.18–0.42	<0.001
Length of follow-up <3 yrs	2277	2468.4	262	10.6	9.4–12.0	1		
Length of follow-up ≥3 yrs^a^	0	3798.1	182	4.8	4.1–5.5	0.37	0.28–0.49	<0.001
Injected within 6 wks	941	1769.2	216	12.2	10.7–14.0	1.50	1.21–1.87	<0.001
Not injected within 6 wks	829	1523.0	126	8.3	6.9–9.9	1		
No response recorded^b^	507	2974.4	102	3.4	2.8–4.2	0.44	0.33–0.57	<0.001
Shared needles within 6 wks	211	331.2	61	18.4	14.3–23.7	1.92	1.46–2.54	<0.001
Not shared within 6 wks	1594	2898.6	281	9.7	8.6–10.9	1		
No response recorded^b^	472	3036	102	3.4	2.8–4.0	0.36	0.29–0.46	<0.001
Unsafe sex within 6 wks	71	227.8	21	9.2	6.0–14.1	1.76	1.12–2.76	0.014
Not had unsafe sex within 6 wks	611	3,625.5	189	5.2	4.5–6.0	1		
No response recorded	1595	2413.2	234	7.0	8.5–11.0	1.73	1.42–2.11	<0.001
NSP low^c^	399	707.8	74	10.5	8.3–13.1	1.19	0.91–1.54	0.199
NSP medium^c^	1059	2622.7	229	8.7	7.6–9.9	1		
NSP high^c^	454	2796.4	114	4.1	3.4–4.9	0.49	0.39–0.61	<0.001
Reports no polydrug use^d^	1847	3230.5	326	10.1	9.1–11.2	0.87	0.57–1.32	0.511
Reports polydrug use^d^	150	277.1	24	8.7	5.8–12.9	1		
No response recorded	280	2758.9	94	3.4	2.8–4.2	0.35	0.28–0.45	<0.001
IDU duration <2 yrs^e^	221	73.4	22	30.0	19.7–45.5	1		
IDU duration 2–<5 yrs^e^	666	752.3	133	17.7	14.9–21.0	0.48	0.30–0.76	0.002
IDU duration 5–<10 yrs^e^	544	2061.5	136	6.6	5.6–7.8	0.17	0.10–0.27	<0.001
IDU duration ≥10 yrs^e^	372	2233.3	85	3.8	3.1–4.7	0.10	0.06–0.17	<0.001
No response recorded	474	1146.0	68	5.9	4.7–7.5	0.16	0.09–0.26	<0.001
Never on OAT during follow-up^f^	1140	2376.4	231	9.7	8.5–11.1	1		
Prior to starting OAT^f^	1036	1117.0	115	10.3	8.6–12.4	1.04	0.83–1.30	0.741
After OAT stopped^f^	0	217.7	19	8.7	5.6–13.7	0.92	0.58–1.47	0.728
Currently on OAT^f^	101	2555.4	79	3.1	2.5–3.9	0.33	0.26–0.43	<0.001

The population size of time-varying factors (including time period, length of follow-up, needle and syringe provision [NSP]) is based on the baseline (at enrolment). For some factors on injecting behaviour, a large number of people with no response were observed; these groups generally had the lowest incidence, aligning with the fact that these risk behaviour questions were asked of PWID that were thought to be higher risk.

a Baseline was client enrolment, and length of follow up at this time was by definition 0 years. At the end of the study, 859 PWID were followed for >3 years.

b 25.1% of PWID were not asked if they had injected within the last 6 weeks, and 45.6% were not asked if they had shared needles in the last 6 weeks.

c Needle and syringe provision (NSP) coverage was defined by calculating the median coverage over 6-month periods between 2012 and 2020. A low coverage period was assigned if the number of syringes distributed was below the lower quartile of the 6-monthly number of syringes distributed over 2012–2020, while a high coverage period was assigned if the number of syringes distributed was above the upper quartile; otherwise, the period was assigned as being medium coverage (see Supplementary Table S1).

d Polydrug use was recorded as ‘yes’ for PWID taking multiple different classes of drugs. This was typically heroin or other opiates plus either amphetamines, yama (a mixture of methamphetamine and caffeine widely used in South East Asia) or alcohol. The majority of PWID in the study reported taking either heroin and/or opiates, which were considered the same drug class and recorded as ‘no polydrug use’.

e Injecting drug use (IDU) duration was calculated by taking the self-reported duration at the first recorded HIV test and adding the length of time followed-up after this.

f Considerable scale-up of OAT was observed throughout the study. By the end of the study, 260 PWID were recorded going onto OAT at a later time point, 81 PWID had stopped taking OAT, and 859 were currently taking OAT. For the analysis of OAT, ‘Prior to starting OAT’ includes all test records of PWID that take OAT during follow-up but have not started yet. ‘After OAT stopped’ includes all test records of PWID that take OAT during follow-up but have now stopped taking OAT. ‘Never on OAT during follow-up’ includes test records of PWID who never took OAT. ‘Currently on OAT’ includes the test records of PWID after their start date of taking OAT and excludes test records of PWID after they discontinued OAT.

### HIV incidence and its risk factors among PWID

**Fig. 1:**
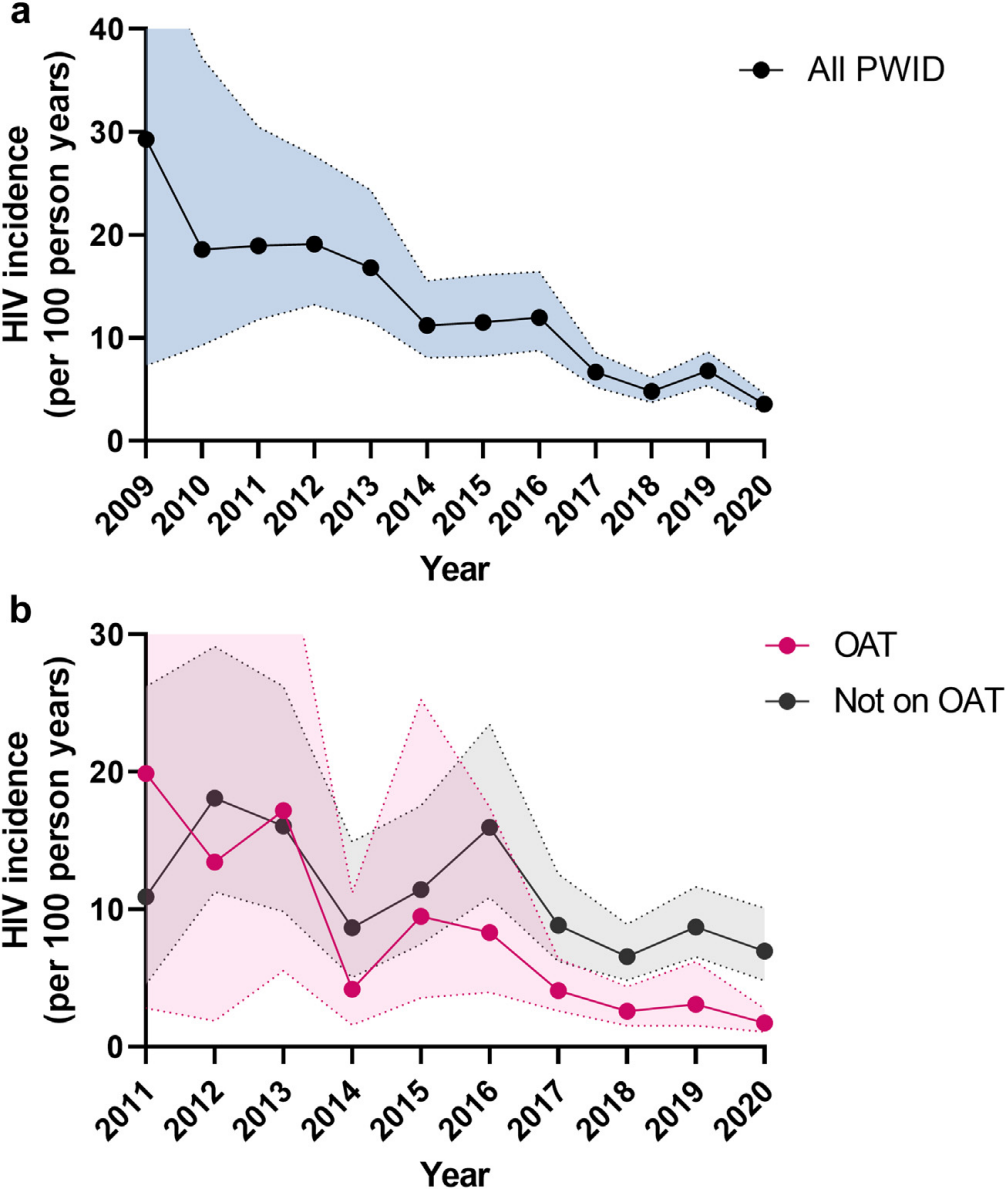
**HIV incidence among (a) PWID and (b) PWID currently on OAT or not on OAT.** HIV incidence was calculated for each year among PWID with 95% confidence intervals indicated by the shaded areas. Data is only presented for 2009 onwards for figure **(a)** and 2011 onwards for figure **(b)** as the low numbers of PWID tested in previous years leads to excessive 95% confidence intervals.

**Fig. 2:**
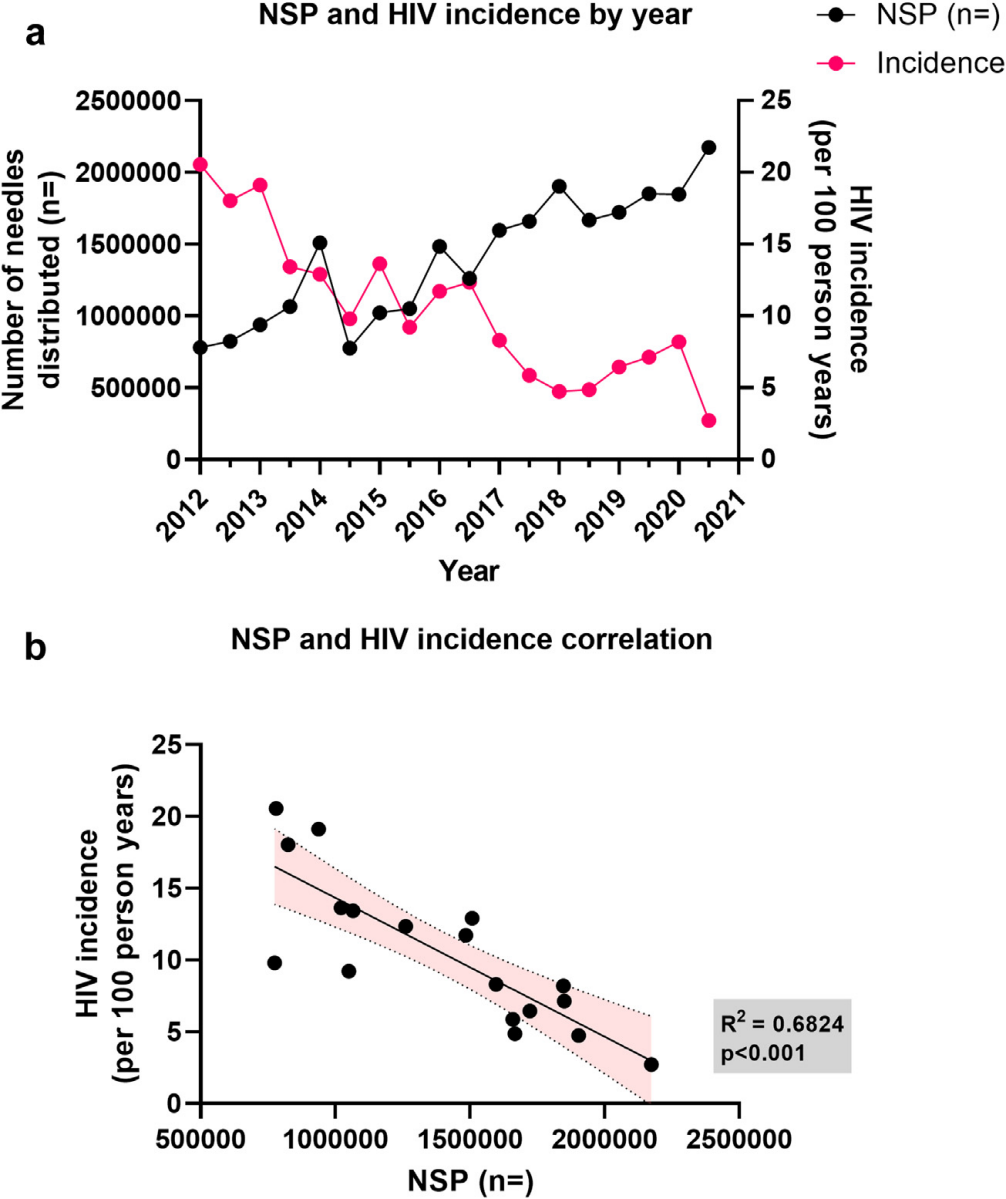
**Relationship between overall 6-monthly needle and syringe provision (NSP) and HIV incidence among PWID across all three drop-in centres, 2012–2020.** (a) NSP coverage is plotted on the left axis (in black) alongside HIV incidence on the right axis (pink) for each 6-month period over 2012–2020. (b) Correlation of NSP coverage and HIV incidence among PWID, using the 6-monthly data points from 2012 to 2020. The shaded area indicates the 95% confidence intervals for the best linear regression model line, with the R^2^ and p-values indicated.

Across the three DIC, the incidence of HIV among PWID declined over time, from 19.3 per 100 py (95% CI 13.3–28.2) over 2008–2011 to 5.2 per 100 py (95% CI 4.6–5.9) over 2017–20 (Table 2 and Fig. 1a). This decrease occurred across all DIC, with overall incidence being higher in Myitkyina (11.4; 95% CI 9.5–13.6) than Hopin (6.5; 95% CI 5.5–7.7) and Mogaung (6.5; 95% CI 5.2–7.0). Unadjusted regression analyses (Table 2 and Fig. 1b) suggest that HIV incidence was reduced by at least half among individuals currently on OAT (HR 0.33 [95% CI 0.26–0.43], p < 0.001, compared to never being on OAT) or during periods of higher syringe coverage (HR 0.49 [0.39–0.61], p < 0.001, compared to medium syringe coverage). Overall, 6-monthly NSP coverage was inversely correlated with HIV incidence (Fig. 2). HIV incidence was also higher among young PWID, female PWID, PWID with shorter injecting durations, those that have recently (last 6 weeks) injected or shared needles, those who report recent unsafe sex and among PWID with shorter follow-up. HIV incidence was generally lowest among PWID not responding to each injecting risk factor question (recent injecting or syringe sharing or polydrug use).

In the full adjusted regression model 1 (Table 3), HIV incidence was estimated to decrease by 15% per year (aHR 0.85 [0.81–0.88], p < 0.001). HIV incidence was 74% higher (aHR 1.74 [1.35–2.25], p < 0.001) among PWID that had recently injected, and 2-fold higher (aHR 2.00 [1.48–2.70], p < 0.001) among PWID that had recently shared needles compared to those that had not. Compared to PWID in the first 2 years of injection drug use, HIV incidence was ∼80% lower among PWID that had injected for 5–10 years (aHR 0.22 [0.13–0.35], p < 0.001) or ≥10 yrs (aHR 0.15 [0.08–0.23], p < 0.001). Sex and polydrug use were not associated with HIV incidence. This regression model could not include NSP and OAT because these exposures were not available for everyone in the dataset.

**Table 3:** Cox regression of factors associated with HIV incidence among the people who inject drugs (PWID) attending the drop-in centres in Kachin, 2008–2020.

Group	Model 1; all sites, 2008–2020			Model 2; HPN and MGG, 2008–2020			Model 3; all sites, 2012–2020			Model 4; HPN and MGG, 2012–2020		
	aHR (adjusted)	95% CI	p-value	aHR (adjusted)	95% CI	p-value	aHR (adjusted)	95% CI	p-value	aHR (adjusted)	95% CI	p-value
Male PWID	1			1			1			1		
Female PWID	1.09	0.54–2.20	0.814	0.78	0.25–2.46	0.670	1.04	0.49–2.21	0.916	0.58	0.18–1.85	0.358
Age <25 yrs^a^	n/a			n/a			n/a			n/a		
Age ≥25 yrs^a^	n/a			n/a			n/a			n/a		
Hopin	1			1			1			1		
Mogaung	0.76	0.56–0.92	0.009	0.82	0.65–1.04	0.103	0.75	0.59–0.96	0.023	0.98	0.77–1.23	0.842
Myitkyina	1.26	0.97–1.65	0.085	n/a			1.21	0.92–1.59	0.178	n/a		
Year of HIV test	0.85	0.81–0.88	<0.001	0.91	0.87–0.95	<0.001	0.88	0.84–0.93	<0.001	0.893	0.88–0.99	0.023
Length of follow-up <3 yrs^a^	n/a			n/a			1			1		
Length of follow-up ≥3 yrs^a^	n/a			n/a			0.58	0.43–0.78	<0.001	0.58	0.41–0.81	0.001
Injected within 6 wks	1.74	1.35–2.25	<0.001	n/a			1.90	1.47–2.46	<0.001	n/a		
Not injected within 6 wks	1			n/a			1			n/a		
No response recorded^b^	1.12	0.59–2.13	0.730	n/a			0.75	0.53–1.06	0.107	n/a		
Shared needles within 6 wks	2.00	1.48–2.70	<0.001	2.37	1.74–3.23	<0.001	n/a			n/a		
Not shared within 6 wks	1			1			n/a			n/a		
No response recorded^b^	0.58	0.31–1.09	0.089	0.43	0.33–0.58	<0.001	n/a			n/a		
NSP medium^c^	n/a			n/a			1			1		
NSP low^c^	n/a			n/a			1.08	0.80–1.45	0.618	0.89	0.63–1.27	0.536
NSP high^c^	n/a			n/a			0.78	0.60–1.01	0.061	0.64	0.48–0.84	<0.001
Reports no polydrug use^d^	1			n/a			1			n/a		
Reports polydrug use^d^	0.98	0.63–1.51	0.924	n/a			1.08	0.70–1.67	0.716	n/a		
No response recorded	0.82	0.59–1.13	0.227	n/a			0.68	0.48–0.97	0.033	n/a		
IDU duration <2 yrs	1			1			1			1		
IDU duration 2–<5 yrs	0.54	0.34–0.86	0.010	0.55	0.31–0.97	0.039	0.45	0.28–0.73	0.001	0.47	0.26–0.83	0.009
IDU duration 5–<10 yrs	0.22	0.13–0.35	<0.001	0.20	0.11–0.35	<0.001	0.20	0.12–0.32	<0.001	0.18	0.10–0.32	<0.001
IDU duration ≥10 yrs	0.14	0.08–0.23	<0.001	0.13	0.07–0.24	<0.001	0.13	0.07–0.21	<0.001	0.11	0.06–0.21	<0.001
No response recorded	0.18	0.11–0.30	<0.001	0.18	0.09–0.33	<0.001	0.16	0.09–0.27	<0.001	0.14	0.08–0.27	<0.001
Never on OAT during follow-up^e^	n/a			1			n/a			1		
Prior to starting OAT^e^	n/a			0.88	0.67–1.15	0.345	n/a			0.87	0.66–1.15	0.324
After OAT stopped^e^	n/a			0.96	0.59–1.57	0.880	n/a			1.08	0.66–1.77	0.769
Currently on OAT^e^	n/a			0.50	0.37–0.68	<0.001	n/a			0.36	0.27–0.48	<0.001

Multiple adjusted models (numbered 1–4) were required as information linking PWID to opioid agonist therapy (OAT) was only available at Hopin (HPN) and Mogaung (MGG), and NSP coverage was available from 2012 to 2020. Model 1 included PWID at all sites but excluding OAT, model 2 analysed the same variables with the addition of OAT, but was restricted to PWID from Hopin (HPN) and Mogaung (MGG). Models 3 (all sites) and 4 (HPN and MGG, including OAT) were similar, but also included NSP coverage with length of follow-up being restricted to 2012–2020. Adjusted Hazard ratios (aHR) are given, with 95% confidence intervals (95% CI) and p-values (p). The reference group is indicated with a value 1, and n/a (not applicable) is used to indicate that analyses in these variables were not included in the model.

a Age was excluded from the regression analysis as it was co-linear with IDU duration. Length of follow up was only included in models 3 and 4 as there were no PWID with <3 yrs of follow up prior to 2012.

b 25.1% of PWID were not asked if they had injected within the last 6 weeks, and 45.6% were not asked if they had shared needles in the last 6 weeks.

c Needle and syringe provision (NSP) coverage was defined by calculating the median coverage over 6-month periods between 2012 and 2020. A low coverage period was assigned if the number of syringes distributed was below the lower quartile of the 6-monthly number of syringes distributed over 2012–2020, while a high coverage period was assigned if the number of syringes distributed was above the upper quartile; otherwise, the period was assigned as being medium coverage (see Supplementary Table S1).

d Polydrug use was recorded as ‘yes’ for PWID taking multiple different classes of drugs. This was typically heroin or other opiates plus either amphetamines, yama (a mixture of methamphetamine and caffeine widely used in South East Asia) or alcohol. The majority of PWID in the study reported taking either heroin and/or opiates, which were considered the same drug class and recorded as ‘no polydrug use’. Polydrug use was excluded from the OAT adjusted models 2 and 4 as polydrug use is very rare in HPN (reported by n = 1).

e For the analysis of OAT, ‘Prior to starting OAT’ includes all test records of PWID that take OAT during follow-up but have not started yet. ‘After OAT stopped’ includes all test records of PWID that take OAT during follow-up but have now stopped taking OAT. ‘Never on OAT’ during follow-up includes test records of PWID who never took OAT. ‘Currently on OAT’ includes the test records of PWID after their start date of taking OAT and excludes test records of PWID after they discontinued OAT.

In reduced adjusted model 2 (Table 3) that just included Mogaung and Hopin DIC, the HIV incidence among PWID that had not yet started OAT (aHR 0.88 [0.67–1.15], p = 0.345) or that had stopped OAT (aHR 0.96 [0.59–1.57], p = 0.880) were comparable to PWID who never took OAT during follow-up. In contrast, the HIV incidence among PWID currently being prescribed OAT was 50% lower (aHR 0.50 [0.37–0.68], p < 0.001).

In reduced adjusted model 3 (Table 3), which only included data for 2012–2020 but all three DIC, higher NSP coverage was borderline associated with reduced HIV incidence (aHR 0.78 [0.60–1.01], p = 0.061, compared to medium syringe coverage). Conversely, in reduced model 4, which included both OAT and NSP (restricted to Mogaung and Hopin and 2012–2020), higher NSP coverage was strongly associated with reduced incidence (aHR 0.64 [0.48–0.84], p < 0.001) as was currently being on OAT (aHR 0.36 [0.27–0.50], p < 0.001, compared to PWID who never took OAT during follow-up). Supplementary Fig. S5 gives the Kaplan–Meier survival curve for HIV incidence among PWID by OAT status showing clearly the lower incidence of infection for those on OAT.

All other associations were similar in reduced models 2, 3 and 4. Recent unsafe sex was not associated with HIV incidence in additional analyses (Supplementary Table S4) Lastly, excluding data from 2020 in reduced model 4 did not affect our findings (Supplementary Table S5), suggesting that closures associated with the SARS-CoV-2 pandemic did not influence observed trends.

## Discussion

Using routinely collected testing and service provision data from three HR interventions in Kachin, Myanmar, we constructed a large longitudinal HIV incidence dataset spanning 12 years. Key findings indicate that PWID in Kachin have high but decreasing HIV incidence, decreasing from 19.3 per 100 py in 2008–2011 to 5.2 per 100 py in 2017–20. This decrease occurred concurrently with the scale-up of OAT and NSP, with our analyses suggesting that being on OAT may have reduced HIV risk by two-thirds, while periods of high NSP coverage may have reduced HIV risk by one-third. Taken together, this evidence suggests that the scale-up of OAT and NSP may have been an important contributor for decreasing HIV incidence among this PWID population in Kachin.

Analyses also showed that new PWID had particularly high HIV incidence, indicating their added vulnerability, while PWID with longer exposure to the DIC had lower incidence. Lastly, PWID had much higher incidence than PWUD (1.0 per 100 py) and their sexual partners (1.1 per 100 py), suggesting that HIV is primarily transmitted through injecting drug use in Kachin.

### Strengths and limitations

The strength of our analysis is the large sample size (3,685) and person years of follow-up (8976.2), paired with considerable incident infections (473). Similar incidence studies in LMIC settings are sparse, with none having similar person years of follow-up or comparable study durations. Our study is also important in being able to estimate the impact of interventions, with no existing studies showing that NSP and OAT can reduce HIV acquisition risk in LMICs.^15^^,^^16^^,^^19^

Limitations include the use of routine testing data collected from individuals attending HR DICs, and so the representativeness of the incidence estimates to the wider PWID population is uncertain. Comparisons of our data to recent bio-behavioural surveys from Myanmar suggest similar levels of syringe sharing, but higher HIV prevalence in our study.^6^ With such a high HIV prevalence at initial testing, it implies substantial ongoing infection is not captured in our estimated incidence rate, potentially biasing our estimates towards a lower incidence rate. Additionally, people that were only tested once could not be included in the incidence dataset. This was the majority of the dataset, with these low return testing rates likely resulting from migration to mining areas and MdM undertaking one-off testing campaigns in difficult-to-access communities in recent years. Our analyses suggest some differences between the full sample of HIV-negative individuals and those that were tested multiple times (Supplementary Table S3), with the main differences being associated with heightened incidence in our analyses, possibly suggesting that we may be overestimating incidence. Lastly, because the study was not a randomised experiment, causal inferences cannot be made about the impact of OAT and NSP.

The dataset was also limited by the small number of questions asked at each HIV test, which is a general limitation of routine datasets. Few questions were included on injecting and sexual risk behaviours, and no questions were asked on structural factors.^20^ Importantly, no data on individual-level NSP was collected, meaning the impact of NSP could only be assessed at the population-level through assessing whether changes in NSP provision were associated with changes in HIV incidence. It is possible that these changes in HIV incidence could be due to changes in other factors. Also, the impact of ART was not evaluated because we were unable to link individuals to whether they had treatment or not. This meant we could not estimate the coverage of ART in the clinic population and determine whether that was associated with reduced incidence. Despite this, we know treatment levels have increased over time, and so this is likely to have contributed to the decline in incidence. A number of factors also had data available only for certain years, or were only asked of individuals thought to have higher risk and so ‘no response’ is frequently associated with lower HIV incidence.

We intended to assess the impact of service disruptions due to the SARS-CoV-2 pandemic, the coup in early-2021 and the ongoing civil war in Kachin. However, the conflict between the KIA and Burmese army caused disruption throughout the study period, making it challenging to identify specific impacts at the sites. Whilst DIC-level data suggests reductions in gate attendance, HIV testing, and people initiating ART and OAT during time-periods related to the SARS-CoV-2 pandemic and the coup (Supplementary Figs. S1, S3 and S4), we could not assess how they impacted on incidence and prevalence, partly due to reduced HIV testing during these periods and uncertainty over what caused changes in prevalence and incidence. Whilst NSP coverage remained consistent throughout these disruptions (Supplementary Fig. S1A), it is possible that these events may have resulted in increased HIV risk among PWID, and so it is important that PWID remain closely monitored and supported during ongoing disruptions.

### Comparison with other studies

This study adds to the small number of HIV incidence estimates among PWID from LMICs. Although initially very high, the overall incidence among PWID (7.1 per 100 py) was lower or comparable to estimates from India (8.0), Pakistan (12.4), and Ukraine (31.9).^21^, ^22^, ^23^ The Kachin cohort (n = 2277 PWID) was larger and covered a longer time-period than other LMIC studies, with only two cohort studies from India (n = 1798)^21^ and Ukraine (n = 589)^23^ including >500 PWID. Studies estimating the impact of OAT on HIV acquisition are sparse in LMICs, with just one previous study from India showing no impact in a lower coverage setting.^21^ Methodological differences may have contributed to these divergent findings as the Indian study analysed the effect of using OAT services in the last 3 months, whereas our study identified time periods when individuals were on OAT, and excluded periods both prior to and after discontinuation of therapy. This may have reduced the magnitude of the association in the Indian study because some people were no longer on OAT. Our finding that OAT reduced HIV risk by approximately two-thirds, whilst high, is comparable to findings from a previous systematic review,^15^ although this only included high income settings. Studies assessing the impact of NSP in LMIC settings are also scarce.^16^ Individual-level data among Indian PWID found that accessing NSP was associated with a higher HIV incidence, although this partly reflected individuals who were injecting more frequently.^21^ Whilst our study was unable to assess NSP access at the individual-level, we did provide evidence that increased NSP coverage may reduce HIV incidence at the population-level.

### Implications

This study highlights the very high incidence of HIV that can occur among PWID in resource poor settings affected by conflict, but also how those incidence levels can still be reduced considerably with concerted scale-up of OAT and NSP. Coverage of these interventions is low globally,^24^ and their clear impact in this study emphasises the need to scale them up in other LMIC settings. Increased scale-up in the region would not have been possible without considerable investment in the peer workforce, and active engagement with local communities to improve acceptance of the interventions. Heightened incidence levels in new injectors also emphasises that they should be a focus of intervention activities. Our study underscores the importance of undertaking detailed routine data collection linked to ongoing interventions, for monitoring trends in HIV incidence and obtaining evidence for the impact of interventions. Similar studies should be undertaken in other settings where comparable data is available, while settings that do not collect such data should consider improving their data collection approaches.

## Contributors

P.V. and E.W. conceived the study. P.V., A.L.M. and J.S. designed the study and methodology. A.L.M. and J.S. developed and A.L.M. performed all data analyses. P.V. supervised the analysis with support from E.W. and J.S. A.L.M., P.V. and J.S. wrote the initial draft of the manuscript. A.L.M. developed the figures. All authors contributed to guiding the overall analysis plan, interpreting the results, and critically reviewing and editing the final version of the manuscript. K.T.O., Z.Z.L., M.T., M.T.A., A.L.M. and E.W. curated the data. J.S. and A.L.M. accessed and verified the data. A.L.M. and P.V. had full access to all the data in the study and had final responsibility for the decision to submit for publication.

## Data sharing statement

The dataset will be made available immediately following publication. The anonymised dataset will be shared with researchers who provide a methodologically sound proposal approved by E.W. and P.V. Proposals should be directed to ernst.wisse@medecinsdumonde.net and peter.vickerman@bristol.ac.uk; requesters will need to sign a data access agreement.

## Declaration of interests

P.V. has received research support from Gilead Sciences, in relation to hepatitis C virus treatment. All other authors declare no conflicts of interest.
