# Supplementary material for: Trends in HIV incidence following scale-up of harm reduction interventions among people who inject drugs in Kachin, Myanmar, 2008–2020: analysis of a retrospective cohort dataset
Source: Lancet Reg Health West Pac. 2023 Feb 27;34:100718. doi: 10.1016/j.lanwpc.2023.100718 (PMC10240370; doi:10.1016/j.lanwpc.2023.100718)
Supplement: Supplementary Tables S1–S5 and Figures S1–S5 [file mmc1.docx]

**Trends in HIV incidence following scale-up of harm reduction interventions among people who inject drugs in Kachin, Myanmar, 2008-2020: Analysis of a retrospective cohort dataset**

**Supplementary information**

**Authors:** McNaughton AL, Stone J, Oo KT, Let ZZ, Taw M, Aung MT, Min AM, Lim AG, Wisse E, Vickerman P

**Tables**

**Supplementary Table 1: Needle and syringe provision over the period 2012-2020, as used to define low, normal and high periods of coverage.** The number of needles distributed is given for each three-month period at each DIC site and across all sites. Median NSP coverage was determined and the lowest 25^th^ percentile used as ‘low’ NSP coverage, and the highest 75^th^ percentile used to define ‘high’ NSP coverage. Green indicates low coverage, red indicates high coverage.

| Year | **Months** | **MKN** | **MGG** | **HPN** |
| --- | --- | --- | --- | --- |
| 2012 | Jan-Jun | 168485 | 235693 | 376342 |
|  | Jul-Dec | 143038 | 291777 | 389654 |
| 2013 | Jan-Jun | 212050 | 335720 | 390220 |
|  | Jul-Dec | 210455 | 467330 | 387832 |
| 2014 | Jan-Jun | 248045 | 705355 | 555413 |
|  | Jul-Dec | 158897 | 369415 | 246783 |
| 2015 | Jan-Jun | 178346 | 445898 | 397016 |
|  | Jul-Dec | 166728 | 492564 | 391432 |
| 2016 | Jan-Jun | 277209 | 566530 | 641651 |
|  | Jul-Dec | 513538 | 240090 | 507983 |
| 2017 | Jan-Jun | 549010 | 291805 | 756774 |
|  | Jul-Dec | 499985 | 213605 | 945904 |
| 2018 | Jan-Jun | 489043 | 445795 | 969108 |
|  | Jul-Dec | 669600 | 311305 | 686863 |
| 2019 | Jan-Jun | 670100 | 463868 | 588905 |
|  | Jul-Dec | 788411 | 568633 | 493420 |
| 2020 | Jan-Jun | 645259 | 599381 | 602795 |
|  | Jul-Dec | 733945 | 725486 | 713962 |

**Supplementary Table 2: Proportions of each patient group included within the non-PWUD and HIV prevalence among those attending their first test.** Proportions of each group considered non-PWUD are provided as a total of the overall dataset (n=20,761), and the HIV prevalence is given in each sub-population.

| **Population** | **Category** | **Frequency (n=)** | **Percent (%)** | **HIV positive (n=)** | **HIV positive (%)** | **95% CI** |
| --- | --- | --- | --- | --- | --- | --- |
| **Children** | Non-PWUD | 341 | 16 | 56 | 16.4 | 12.8-20.8 |
| **Partner of person who injects drugs (PWID)** |  | 2,052 | 9.9 | 333 | 16.2 | 14.7-17.9 |
| **Partner of person who uses drugs (PWUD)** |  | 847 | 4.1 | 100 | 11.8 | 9.8-14.2 |
| **General population** |  | 133 | 0.6 | 3 | 2.3 | 0.7-6.8 |

**Supplementary Table 3; Demographic information on HIV negative PWID cases in Myanmar, 2008-2020**. From a total of 20,791 DIC attendees, 13,057 were HIV-negative at their first test with 3,685 attendees recording more than one HIV test, making them eligible for inclusion in the incidence analysis. Baseline characteristics of all the HIV-negative cases are compared with those included in the incidence dataset.

| **HIV status and cohort** | |  | **HIV negative (prevalence cases)** | | **HIV negative (incidence cases)** | | |  | |
| --- | --- | --- | --- | --- | --- | --- | --- | --- | --- |
|  |  | Total | **n=** | **(%)^a^** | | **(%)^a^** | **(%)^a^** | | **p^b^** |
|  | All | 20,791 | 13,057 | 62.9 | | 3,685 | 100 | | **-** |
| **DIC client group** | PWID | 14,069 | 7,259 | 55.6 | | 2,277 | 61.8 | |  |
|  | PWUD | 3,352 | 2,936 | 22.5 | | 692 | 18.8 | |  |
|  | non-PWUD | 3,340 | 2,862 | 21.9 | | 716 | 19.4 | | <0.001 |
| **Among PWID only** | |  |  |  | |  |  | |  |
| **Gender** | Male | 13,849 | 7,155 | 98.6 | | 2,271 | 98.7 | |  |
|  | Female | 220 | 104 | 1.4 | | 31 | 1.3 | | 0.426 |
| **Age** | Mean (yrs) | 30.1 (CI 30.0-30.2) | 29.9 | (CI 29.7-30.1) | | 29.0 | (CI 28.6-29.3) | | <0.001 |
| **Centre** | Hopin | 4,343 | 2,160 | 29.8 | | 773 | 33.9 | |  |
|  | Mogaung | 3,871 | 2,160 | 29.8 | | 962 | 42.2 | |  |
|  | Myitkyina | 5,855 | 2,939 | 40.5 | | 542 | 23.8 | | <0.001 |
| **Year of test** | 2008-11 | 1,532 | 838 | 11.5 | | 365 | 16.0 | |  |
|  | 2012-16 | 4,526 | 2,324 | 32.0 | | 975 | 42.8 | |  |
|  | 2017-20 | 8,011 | 4,097 | 56.4 | | 937 | 41.2 | | <0.001 |
| **Injected drugs within last 6 weeks** | No | 4,205 | 2,182 | 30.1 | | 829 | 36.4 | |  |
|  | Yes | 6,177 | 3,178 | 43.8 | | 941 | 41.3 | |  |
|  | No response | 3,687 | 1,899 | 26.2 | | 507 | 22.3 | | <0.001 |
| **Shared needles within last 6 weeks** | No | 9,056 | 4,909 | 67.6 | | 1,594 | 70.0 | |  |
|  | Yes | 1,433 | 505 | 7.0 | | 211 | 9.3 | |  |
|  | No response | 3,580 | 1,845 | 25.4 | | 472 | 20.7 | | <0.001 |
| **Unsafe sex within last 6 weeks** | No | 4,671 | 2,440 | 33.6 | | 611 | 26.8 | |  |
|  | Yes | 558 | 293 | 4.0 | | 71 | 3.1 | |  |
|  | No response | 8,840 | 4,526 | 62.4 | | 1,595 | 70.0 | | <0.001 |
| **Reports polydrug use** | No^b^ | 11,069 | 5,716 | 78.7 | | 1,847 | 81.1 | |  |
|  | Yes | 682 | 355 | 4.9 | | 150 | 6.6 | |  |
|  | No response | 2,318 | 1,188 | 16.4 | | 280 | 12.3 | | <0.001 |
| **Age at first use** | mean (yrs) | 24.6 (CI 24.4-24.7) | 24.7 | (CI 24.5-24.9) | | 23.1 | (CI 22.9-23.5) | | <0.001 |
| **Duration of IDU** | mean (yrs) | 5.9 (CI 5.8-5.9) | 5.5 | (CI 5.4-5.7) | | 6.0 | (CI 5.7-6.2) | | <0.001 |

**^a^** For age, age at first drug use and duration of injection drug use (IDU), the 95% confidence interval of the mean is provided in this column. For each factor, the proportions in each group have been calculated based on the total number of PWID in each group.

**^b^**Statistical significance was assessed using multiple contingency tables and Kruskal–Wallis tests as appropriate.

**Supplementary Table 4: Adjusted Cox regression of factors associated with HIV incidence among the population and PWID in Myanmar from 2017-2020 to investigate recent unsafe sex.** The adjusted models presented in this table include models 5 and 6, which are similar to models 1 and 2 (Table 3) but also include data on unsafe sex and are restricted to 2017-2020. Hazard ratios (aHR) are given, with 95% confidence intervals (95CI) and p-values (p). The reference group, is indicated with a value 1, and n/a (not applicable) is used to indicate that analyses in these variables were not included in the model.

| **Group** | **HR (adjusted)** | **95 CI** | **p** | **HR (adjusted)** | **95 CI** | **p** |
| --- | --- | --- | --- | --- | --- | --- |
| **Among PWID only** | *(NSP model 5 - all sites)* | | | *(NSP model 6 - HPN and MGG only)* | | |
| **Male PWID** | 1 |  |  | 1 |  |  |
| **Female PWID** | 0.71 | 0.182.91 | 0.640 | 0.38 | 0.51-2.72 | 0.333 |
| **Age <25yrs** | n/a |  |  | n/a |  |  |
| **Age ≥25yrs** | n/a |  |  | n/a |  |  |
| **Hopin** | 1 |  |  | 1 | | |
| **Mogaung** | 0.88 | 0.64-1.22 | 0.450 | 0.90 | 0.66-1.24 | 0.526 |
| **Myitkyina** | 1.04 | 0.71-1.53 | 0.835 | n/a |  |  |
| **Year of test** | 0.82 | 0.72-0.93 | 0.002 | 0.87 | 0.76-1.00 | 0.45 |
| **Injected within 6wks** | 2.5 | 1.73-3.65 | <0.001 | n/a |  |  |
| **Not injected within 6wks** | 1 |  |  | n/a |  |  |
| **No response recorded** | 0.91 | 0.34-2.42 | 0.850 | n/a |  |  |
| **Shared needles within 6wks** | 1.96 | 0.36-2.83 | <0.001 | 2.37 | 1.62-3.48 | <0.001 |
| **Not shared within 6wks** | 1 |  |  | 1 |  |  |
| **No response recorded** | 1.05 | 0.41-2.69 | 0.916 | 0.42 | 0.29-0.60 | <0.001 |
| **Unsafe sex within last 6 weeks** | 1.30 | 0.81-2.06 | 0.274 | 1.31 | 0.75-2.30 | 0.341 |
| **No unsafe sex with 6wks** | 1 |  |  | 1 |  |  |
| **No response recorded** | 0.92 | 0.65-1.30 | 0.622 | 0.70 | 0.46-1.06 | 0.091 |
| **Reports no polydrug use^a^** | 1 |  |  | n/a |  |  |
| **Reports polydrug use^a^** | 0.79 | 0.48-1.29 | 0.351 | n/a |  |  |
| **No response recorded** | 0.92 | 0.32-1.03 | 0.064 | n/a |  |  |
| **IDU duration <2yr** | 1 |  |  | 1 |  |  |
| **IDU duration 2-<5yrs** | 0.42 | 0.22-0.81 | 0.009 | 0.34 | 0.17-0.68 | 0.003 |
| **IDU duration 5-<10yrs** | 0.20 | 0.05-0.20 | <0.001 | 0.09 | 0.04-0.20 | <0.001 |
| **IDU duration ≥10yrs** | 0.07 | 0.04-0.15 | <0.001 | 0.07 | 0.03-0.14 | <0.001 |
| **No response recorded** | 0.10 | 0.05-0.20 | <0.001 | 0.09 | 0.04-0.20 | <0.001 |
| **Never on OST** | n/a |  |  | 1 |  |  |
| **Prior to starting OST** | n/a |  |  | 0.87 | 0.60-1.24 | 0.436 |
| **After OST stopped** | n/a |  |  | 1.06 | 0.62-1.82 | 0.838 |
| **Currently on OST** | n/a |  |  | 0.52 | 0.36-0.75 | <0.001 |

**^a^** Polydrug use was recorded as ‘yes’ for PWID taking multiple different classes of drugs. This was typically heroin or other opiates plus either amphetamines, yama (a mixture of methamphetamine and caffeine widely used in South East Asia) or alcohol. The majority of PWID in the study reported taking either heroin and/or opiates, which were considered the same drug class and recorded as ‘no polydrug use’.

**Supplementary Table 5: Adjusted Cox regression of factors associated with HIV incidence among PWID in Myanmar from 2012-2019.** The adjusted model presented in this table is a modified version of ‘model 4’ (presented in manuscript, Table 3) with the year of the SARS-CoV-2 outbreak (2020) excluded from the analysis. Adjusted hazard ratios (aHR) are given with 95% confidence intervals (95%CI) and p-values. The reference group is indicated with a value 1, and n/a (not applicable) is used to indicate that analyses for these variables were not included in the model.

|  | **Model 4** *with 2020 excluded* | | |
| --- | --- | --- | --- |
| **Group** | **aHR (adjusted)** | **95%CI** | **p-value** |
| **Male PWID** | 1 |  |  |
| **Female PWID** | 0.76 | 0.23-2.46 | 0.652 |
| **Age <25yrs** | n/a |  |  |
| **Age ≥25yrs** | n/a |  |  |
| **Hopin** | 1 |  |  |
| **Mogaung** | 0.91 | 0.70-1.17 | 0.468 |
| **Myitkyina** | n/a |  |  |
| **Year of HIV test** | 0.94 | 0.87-1.00 | 0.055 |
| **Length of follow-up <3yrs** | 1 |  |  |
| **Length of follow-up ≥3yrs** | 0.56 | 0.39-0.80 | 0.002 |
| **Injected within 6wks** | n/a |  |  |
| **Not injected within 6wks** | n/a |  |  |
| **No response recorded** | n/a |  |  |
| **Shared needles within 6wks** | n/a |  |  |
| **Not shared within 6wks** | n/a |  |  |
| **No response recorded** | n/a |  |  |
| **NSP medium^a^** | 1 |  |  |
| **NSP low^a^** | 0.90 | 0.63-1.29 | 0.567 |
| **NSP high^a^** | 0.64 | 0.46-0.89 | 0.008 |
| **Reports no polydrug use^b^** | n/a |  |  |
| **Reports polydrug use^b^** | n/a |  |  |
| **No response recorded** | n/a |  |  |
| **IDU duration <2yr** | 1 |  |  |
| **IDU duration 2-<5yrs** | 0.47 | 0.25-0.87 | 0.016 |
| **IDU duration 5-<10yrs** | 0.18 | 0.10-0.35 | <0.001 |
| **IDU duration ≥10yrs** | 0.13 | 0.07-0.26 | <0.001 |
| **No response recorded** | 0.15 | 0.07-0.29 | <0.001 |
| **Never on OAT during follow-up** | 1 |  |  |
| **Prior to starting OAT** | 0.94 | 0.70-1.27 | 0.701 |
| **After OAT stopped** | 1.19 | 0.69-2.09 | 0.528 |
| **Currently on OAT^e^** | 0.40 | 0.29-0.57 | <0.001 |

**^a^** Needle and syringe provision (NSP) was defined by calculating the median coverage over 6-month periods. The lower quartile periods were considered ‘low’ and upper quartile considered ‘high’, with all other periods considered ‘medium’(see Supplementary Table 1).

**^b^** Polydrug use was recorded as ‘yes’ for PWID taking multiple different classes of drugs. This was typically heroin or other opiates plus either amphetamines, yama (a mixture of methamphetamine and caffeine widely used in South East Asia) or alcohol. The majority of PWID in the study reported taking either heroin and/or opiates, which were considered the same drug class and recorded as ‘no polydrug use’.

**Figures**

**Supplementary Figure 1; (A) Mean number of monthly needles distributed and (B) mean number of monthly HIV tests and new antiretroviral (ART) and opioid agonist therapy (OAT) prescriptions at each DIC.** Means were averaged from data at Myitkyina and Mogaung from 2008-2010, and from all three DICs including Hopin for 2011-2020. Monthly provision of needles was only available from 2012 onwards. OAT was only available at Mogaung and Hopin. Graphs were smoothed by taking an average including the two months either side of each plotted value. Further details, showing coverage at each DIC is plotted in Supplementary Figure 3. The increase in testing in 2017 reflects an increase in the testing undertaken by peer workers, including an increased focus on undertaking testing in new areas and new populations.

**Supplementary Figure 2; Prevalence of HIV at first HIV test in (A)** **all persons who inject drugs (PWID), persons who use drugs (PWUD) and persons not using drugs (non-PWUD) in all three drop-in centres, and** **(B) all PWID at Myitkyina (MKN), Mogaung (MGG) and Hopin (HPN) drop-in centres over 2008-2020.** Data from Hopin was only available from 2011 onwards. The proportion of attendees testing HIV-positive at their first test is shown for each year, with 95% confidence intervals.

**Supplementary Figure 3; Numbers of (A) needles distributed, (B) people tested and (C) initiated on antiretroviral therapy (ART) or (D) opiate substitution therapy (OST) in Hopin (HPN), Mogaung (MGG) and Myitkyina (MKN) over 2008-2020.** Estimates for number of needles distributed were only available from January 2012. OST was not used as an intervention at the MKN site by Médecins du Monde. Closure of the MGG DIC site by community resistance in 2016/17 can be seen to impact HIV testing, NSP, and ART initiation. OAT services in the area were managed at a separate site at the local hospital compound, meaning they were able to continue without major disruptions.

**Supplementary Figure 4; Monthly number of attendees at DICs in Hopin (HPN), Mogaung (MGG), and Myitkyina (MKN) over October 2011- August 2021**. This was provided as head count data, meaning the same individual may be counted in the data multiple times if they attended the DIC multiple times within the month. Attendees are stratified by patient group, which includes people who inject drugs (PWID), people who use drugs (PWUD), partners of both PWID (PWID-P) and PWUD (PWUD-P), as well as children (CH) and the general population (GP).


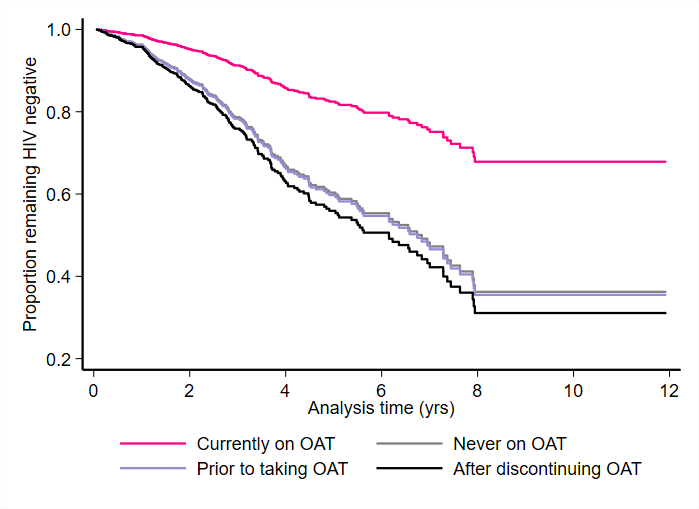


**Supplementary Figure 5; Kaplan-Meier survival curve for HIV infection risk among PWID whilst on and off OAT.** Survival analysis is adjusted for cox regression analysis (based on ‘Model 4’, Table 3). Year of HIV test was excluded from the model in order to plot the graph.
